# WHAT WAS IMPORTANT TO OLDER VOTERS IN THE 2020 ELECTION? A LOOK AT COVID-19, MISINFORMATION, AND POLICY

**DOI:** 10.1093/geroni/igac059.135

**Published:** 2022-12-20

**Authors:** Elizabeth Rickenbach, Janelle Fassi, Kevin Doran

**Affiliations:** St. Anselm College, Manchester, New Hampshire, United States; University of Massachusetts Boston, Goffstown, New Hampshire, United States; st. Anselm, Manchester, New Hampshire, United States

## Abstract

High older voter turnout rates, a growing aging population, and organizations that serve the interests of older adults have historically contributed to the importance of older adults for elections. Since 2010 older voters have tended to vote Republican, with White older adults typically preferring Republican candidates, and Black and Hispanic older voters typically preferring Democratic candidates. In the 2020 election, the 65+ group of voters showed the same Republican candidate favorability but followed a slow downward trend from recent elections. Grounded in a demographic, economic, and generational context, and considering theory and research from gerontology, political science, psychology, and sociology, this presentation will explore older voter turnout and candidate choice in the 2020 presidential election. The focus will be on considering past trends and public polling data to examine three key issues in relation to the behavior of older voters in 2020: COVID-19, presidential candidate platforms, and misinformation and the media.

